# Early cancer detection using the fluorescent Ashwagandha chitosan nanoparticles combined with near-infrared light diffusion characterization: in vitro study

**DOI:** 10.1007/s10103-022-03678-x

**Published:** 2023-01-11

**Authors:** Hala S. Abuelmakarem, Omnia Hamdy, Mahmoud A. Sliem, Jala El-Azab, Wafaa A. Ahmed

**Affiliations:** 1 System and Biomedical Engineering Department, The Higher Institute of Engineering, El Shoruk Academy, El-Shorouk, Egypt; 2 Department of Engineering Applications of Lasers, National Institute of Laser Enhanced Sciences (NILES), Cairo University, Giza Governorate, Giza, 12613 Egypt; 3 Department of Laser Applications in Metrology, Photochemistry and Agriculture (LAMPA), National Institute of Laser Enhanced Sciences (NILE), Cairo University, Giza, 12613 Egypt; 4 Cancer Biology Department, Biochemistry and Molecular Biology Unit, National Cancer Institute, Cairo University, Giza, Egypt; 5 Chemistry Department, Faculty of Science, Taibah University, Al-Ula, Medina, Saudi Arabia

**Keywords:** Colon cancer, NIR laser, Ashwagandha chitosan nanoparticles, Light diffusion

## Abstract

Early cancer diagnosis through characterizing light propagation and nanotechnology increases the survival rate. The present research is aimed at evaluating the consequence of using natural nanoparticles in cancer therapy and diagnosis. Colon cancer cells were differentiated from the normal cells via investigating light diffusion combined with the fluorescence effect of the Ashwagandha chitosan nanoparticles (*Ash C NPs*). Ionic gelation technique synthesized the *Ash C NPs*. High-resolution transmission electron microscope, dynamic light scattering, and zeta potential characterized *Ash C NPs*. Fourier transform infrared spectroscopy analyzed *Ash C NPs*, chitosan, and Ashwagandha root water extract. Moreover, the MTT assay evaluated the cytotoxicity of *Ash C NPs* under the action of near-infrared light (NIR) irradiation. The MTT assay outcomes were statistically analyzed by Bonferroni post hoc multiple two-group comparisons using one-way variance analysis (ANOVA). Based on the Monte-Carlo simulation technique, the spatially resolved steady-state diffusely reflected light from the cancerous and healthy cells is acquired. The diffuse equation reconstructed the optical fluence rate using the finite element technique. The fluorescent effect of the nanoparticles was observed when the cells were irradiated with NIR. The MTT assay revealed a decrease in the cell viability under the action of *Ash C NPs* with and without laser irradiation. Colon cancer and normal cells were differentiated based on the optical characterization after laser irradiation. The light diffusion equation was successfully resolved for the fluence rate on cells’ surfaces showing different normal and cancer cells values. *Ash C NPs* appeared its fluorescent effect in the presence of NIR laser.

## Introduction

Colon cancer is a prevalent cause of death internationally. It is characterized by abnormal growth of precancerous polyps causing colorectal cancer in the colon or rectum [1]. According to the disease control and prevention centers, about 53 k colon cancer deaths were recorded in 2021 [2]. However, early tumor detection increases the healing probabilities and advances tumor management according to the cancer stage. Early screening tests can detect colorectal cancer at its initial stage and advance the healing levels [3]. Medical screening technology plays a chief role in early cancer detection to enhance the therapeutic chances and decrease the mortality rate [4]. The traditional contrast agents (luminescent materials such as quantum dots and organic dyes) utilized in cancer detection have some limitations compared with natural nanophotonic materials that have high optical stability, chemical stability, and narrow band gap emission [5, 6].

The nanoparticles used in nanomedicines are small-sized carrier materials encapsulating imaging and/or therapeutic antioxidant compounds used for theranostic issues [7]. Recently, medical nanoparticles have been used to overcome the body’s biological barriers to improve the delivery of theranostic nanoparticles to specific tissues and tumor lesions [8]. The significant feature of the diagnostic nanoparticles is determining the biodistribution of the nanoparticles in the molecular cells; this aims to enhance the medical tomography and differentiate normal from abnormal lesions. In particular, the enhanced permeability and retention effect leads to nanoparticle accumulation in cancer lesions [8].

Synthesizing nanoparticles from natural materials is a safe technique, especially for theranostic purposes. Ashwagandha (Withania somnifera) is a familiar herbal medicine that has an antitumor, anti-inflammatory, and antioxidant effect [9, 10].

The Withania somnifera root extract (WSRE) inhibits cancer cells grow because of the steroidal lactones and alkaloids [11, 12]. Withania somnifera cytotoxicity has also been evaluated against cancer cells [13], and its therapeutic effect in traditional therapy has been assessed [12]. Chitosan is also a platform for tumor therapy and medical imaging applications because it has active functional groups and appears as shining light [14, 15].

Chitosan has approval from the European Medicine Agency and the Food & Drug Administration (FDA) as a non-toxic polymer [16–18]. Chitosan is an amino polysaccharide molecule with positive electrostatic charges that attract negative molecule charges. Thus, chitosan allows the stability of the nanoparticles inside the nanosolution due to the amino groups [19], which allows ionic interaction with multivalent elements [20]. Several studies reveal the significance of chitosan in medical optical diagnosis [21, 22], drug-carrying [23], molecular imaging [18], gene delivery [24], and tumor treatment [25]. For the therapeutic purpose, Abulemakarem HS et al. [25] demonstrated the role of chitosan nanoparticles in cancer therapy against the Caco2 cell line in vitro. Loutfy AS et al. [26] assessed the cytotoxicity of chitosan NPs against human liver carcinoma in vitro.

Additionally, Queiroz MF et al. [27] demonstrated other therapeutic effects of chitosan NPs. Yang SJ [28] formed nanoparticles from folic acid-modified chitosan and alginate aimed at detecting colorectal cancer via the fluorescent endoscopic. Zhu H-Z et al. [29] detected the lung cancer cells using chitosan NPs. Nanoparticles have superior absorption properties and allow photothermal therapy utilization [8]. The upconversion NPs were assessed in *vitro* and in *vivo* using three modes of imaging function (UCL/fluorescence/magnetic resonance) to evaluate the theranostic effect using 808 and 980 near-infrared light irradiations. The synthesized NPs caused burns in the skin during irradiation with 980 nm, when the lesions were irradiated for 5 min [30]. Thus, engineers and scientists directed to the photonic natural NPs.

Near-infrared light irradiation has a deep biological tissue penetration. Therefore, it has been widely used in biological and biomedical applications [31, 32]. Moreover, the enhanced scattering properties of nanoparticles as near-infrared (*NIR*) contrast agents promise safely nonionizing imaging due to the low scattering and absorption properties. Previous research proved the role of the combination between the nanoparticles and the *NIR* in cancer theranostic [15]. The previous research applied the effect of the photo modulation therapy by converting the laser beam into heat under the control of the nanotubes (CNTs) and titanium dioxide (TiO_2_) nanoparticles against the melanoma tumor sites of cancerous mice because those nanomaterials have specific absorption and scattering properties [33]. Other research coated the oxidized CNTs with silver nanoparticles and the polyethylene glycol to study their photothermal effect against malignant tumors under near-infrared radiation at 808 nm. They proved that their synthesized nanoparticles are a good candidate to root out malignant tumors using the PTT method [34].

Additionally, chitosan NPs promised excellent imaging in exploring the biological systems and early disease detection [35]. Recently, ultra-small nanoparticles conjugated to a melanoma biomarker are utilized as a contrast agent in optical coherence tomography (OCT), showing improvement in differentiating melanoma cells from nonmelanoma cell differentiation in vitro [36]. Diffuse optical tomography is a safe, non-invasive technology that employs a laser in the red-*NIR* region (600 to 900 nm) to probe biological tissues/organs and characterize their absorption and scattering properties [37–40]. These properties control the light propagation in the observed tissues and differ according to the applied wavelength [41–43].

Furthermore, tissue conditions affect its absorption and scattering characteristics. Therefore, these parameters can be utilized in tissue monitoring [44] and medical diagnosis [45, 46]. The technique is called “diffuse” because light propagation in tissues at that optical region follows a diffusion process due to light’s dense and multiple scattering behavior [47, 48]. Determining absorption and scattering parameters requires knowledge of tissue reflectance and transmittance, which can be measured using either an integrating sphere or a distant detector [49, 50]. The optical parameters were estimated based on the measured diffused light [51].

In the current study, Ashwagandha chitosan nanoparticles (*Ash C NPs*) were synthesized and characterized to evaluate their theranostic effect combined with the diffuse optical imaging method. Cancer cells have been differentiated from normal cells based on their optical tissue parameters. The differentiation criterion depends on the fluence distribution photographs at the tissue surface with and without the usage of the *Ash C NPs*. Results evaluate the fluorescent light on the cells’ surface injected with *Ash C NPs* and irradiated with *near-infrared* during the cells imaging. Moreover, the cytotoxicity of the *Ash C NPs* was evaluated against colon carcinoma in vitro (Caco2 cells) when the cells were irradiated by NIR and without NIR irradiation. The *Ash C NPs* are suitable candidates for theranostic applications in vivo in preoperative localization of cancer and intra-operative management.

The scientific research is organized as the following, the first section introduces the scientific idea, including the objectives and organization. The second section represents the methodology which includes the preparation and characterization of the nanoparticles, the cell culture, and therapeutic and diagnostic assay, including the optical parameter determination and the modeling of the optical fluence distribution and the observing the fluorescent effect of the Ashwagandha chitosan NPs during cells imaging. The obtained results and the discussion from this work are represented in the third section, followed by the conclusions.

## Methods

Egyptian Ashwagandha root water extract (*Ash*) was purchased from the National Cancer Institute (NCI), Cairo, Egypt. Chitosan (degree of deacetylation: 75%, molecular weight: 161.1 MW) was obtained from Oxford Company, India. Semiconductor CW laser diodes at 665 nm and 808 nm were obtained from the Laser Land Company, China.

### Nanoparticle synthesizing

**Fig. 1:**
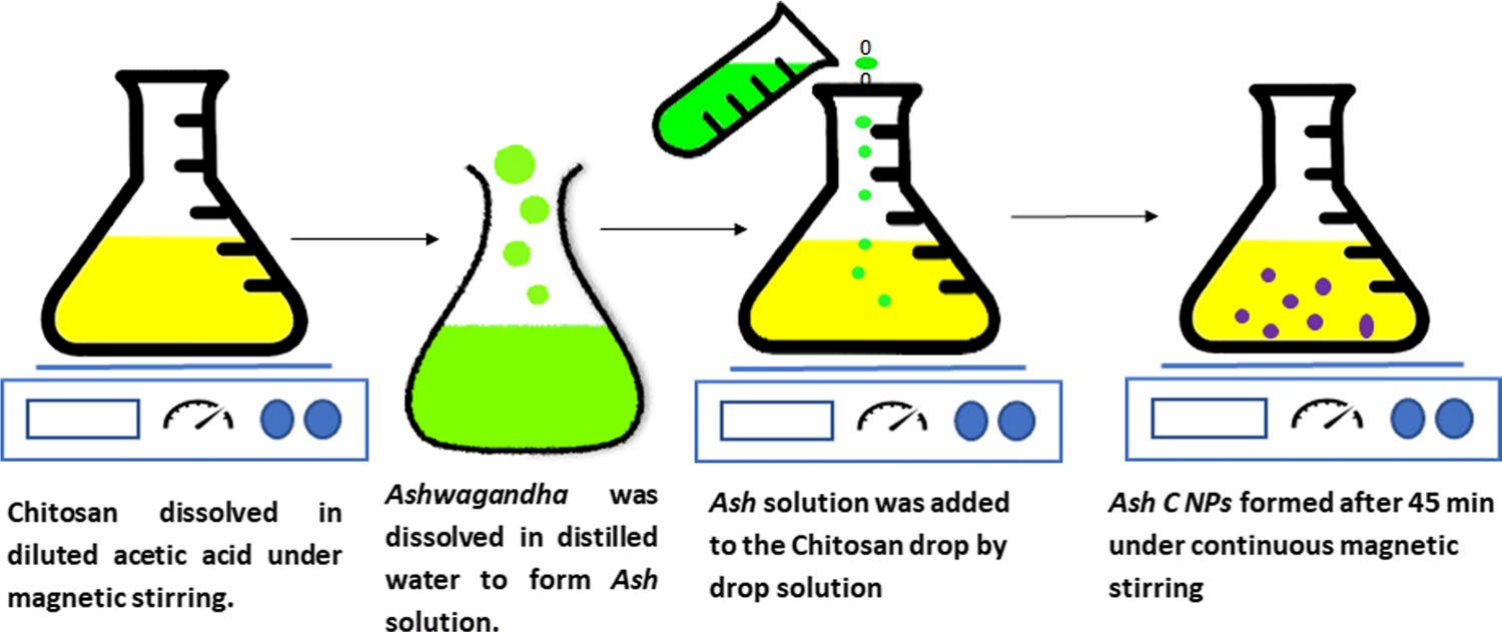
Ashwagandha chitosan nanoparticle synthesizing stages

The Ashwagandha chitosan nanoparticles (*Ash C NPs*) were prepared in four steps—as shown in Fig. 1—as follows: first, chitosan (150 mg) was dissolved in diluted acetic acid (150 mL) for 1.5 h under the action of continuous magnetic stirring. Second, 50 mg *of Ash* was dissolved in 10 mL of 1% distilled water to form *Ashwagandha (Ash)* solution.

Third, Ash solution was added to the chitosan solution drop by drop. Fourth, *Ash C NPs* formed after 45 min under the action of 2000 rpm of continuous magnetic stirring at room temperature. *Ash C NP* solution was dialyzed using a centrifuge at 12,000 rpm.

### Nanoparticle characterization

The *Ash C NPs* were characterized using Fourier transform infrared spectroscopy (FT/IR) to determine the functional groups. Dynamic light scattering (DLS) measured the hydrodynamic particle size, while the zeta potential (ZP) observed the stability of the nanoparticles. High-resolution electronic transmission microscope (HR-TEM) provided the morphologic, crystallographic information and compositional of the *Ash C NPs*.

#### Fourier transform infrared spectroscopy analysis

The *natural components of the Ash C NPs* were analyzed by analyzing the spectra of the *FTIR*. It was employed to carry out the infrared (IR) spectra for each of the chitosan, *Ash*, and *Ash C NPs* to detect their chemical functional groups and to study their capping ligands. The analyzed device, provided by FT/IR-400, JASCO, Tokyo, Japan, has a triglycine sulfate detector (TGS). The scanning transmission was attuned at scanning speed, with a spectral range of two mm/s, 4000–500 cm^−1^, respectively.

#### Dynamic light scattering and zeta potential measurements

Dynamic light scattering (DLS) was employed to measure the hydrodynamic size and the potential charge of the *Ash C NPs*. The DLS measurement device was ZS-ZEN (Malvern Instruments Co., UK). It has a measurement range from 0.6 to 6000 nm and a zeta potential range of − 200:200 mV. The DLS measurement was at room temperature with a scattering angle of 173°, the particle diameter size distribution of the *ASh C NPs*, and their surface charge.

#### High-resolution transmission electron microscope imaging

The high-resolution transmission electron microscope (HR-TEM, Tecnai G20, FEI, Netherlands) was used for imaging purposes. Two different imaging modes were employed: the bright field at electron accelerating voltage 200 kV using lanthanum hexaboride (LaB6) electron source gun and the diffraction pattern imaging. Eagle CCD camera with (4 k*4 k) image resolution was used to acquire and collect transmitted electron images. TEM Imaging & Analysis (TIA) software was used for spectrum acquisition and analysis of EDX peaks. The TEM was applied in two steps: first, *ASh C NP* samples were pipetted up and down to be suspended, second, 2–5 μL drops of samples were mounted on carbon-coated 400-mesh copper grids, and the specimens were left to dry for 2 min. The filter paper was used to remove excess solution and facilitate the settling down of particles on the grid.

### Cell line

Caco-2 cell line (colon carcinoma) and WI-38 cell line (normal human lung epithelial tissue) were obtained from the NCI, Cairo, Egypt. In few lines, the cells were cultured in a T-75 flask using RPMI 1640 medium (Sigma-Aldrich, St. Louis, MO, USA). Then, they were incubated, and the medium’s well was removed. After the cells maintaining in RPMI 1640 medium for 24 h, the cells were washed two times using 1.0 M phosphate-buffered saline (PBS; pH 7.4), then the medium was replaced with *Ash C NPs*.

### Discrimination of cancerous cells using Ash C NPs and NIR laser light propagation

The experiment was applied to differentiate cancerous cells (Caco-2 cells) from healthy cells (WI-38 cells) using the optical tissue parameters and the fluence rate distribution and to observe the fluorescence light of the *Ash C NPs* on the Caco-2 cells’ surface during *NIR* laser exposure.

**Fig. 2:**
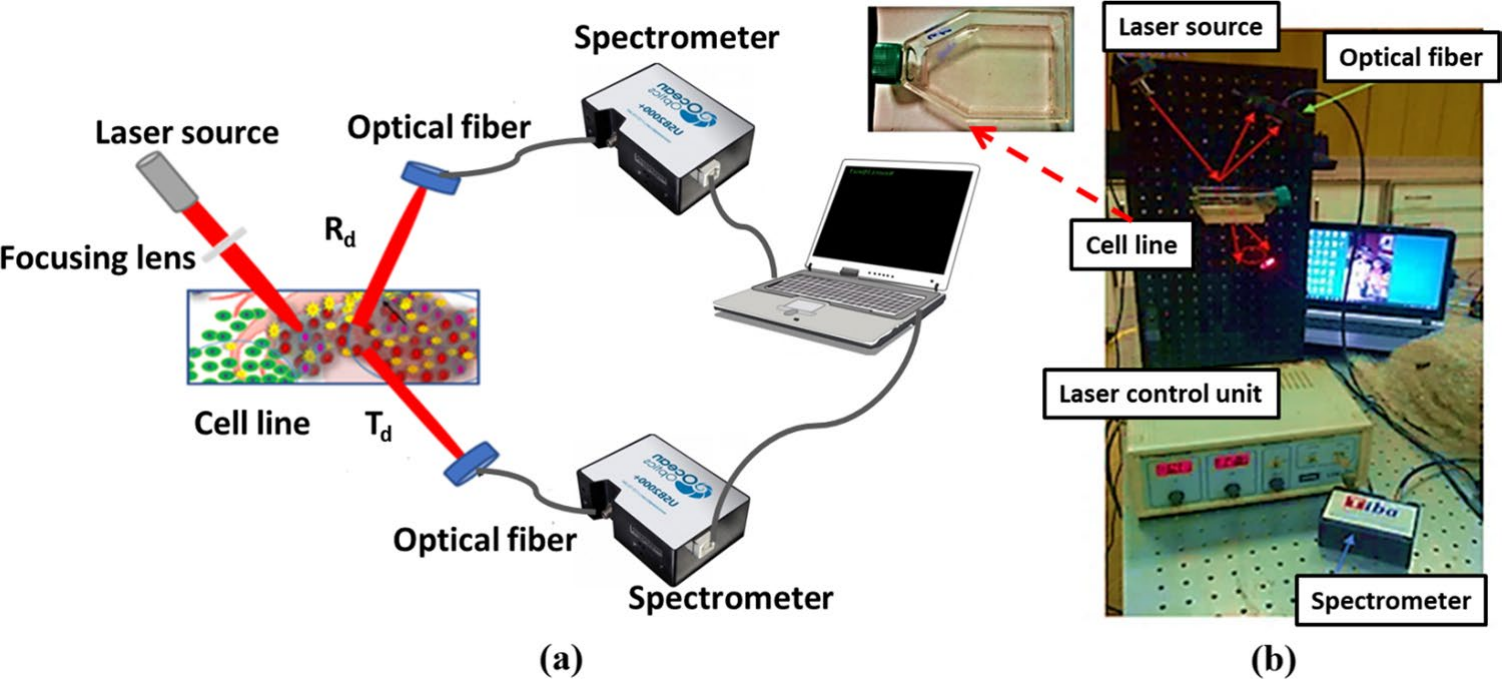
**a** Schematic of the optical setup for measuring reflectance and transmittance; **b** lab photo of the setup

Semiconductor laser diodes at wavelengths (665, 808 nm) were used to probe the cell lines. Figure 2 represents a schema of the optical setup to distinguish the cancer cells from the healthy cells based on the diffuse optics method. The incident laser source was focused on the cells, and the transmitted and reflected light was collected out of a fiber optic (STDFSM, Touptek Photonics Co. Ltd., Zhejiang China) to be analyzed through a compact spectrometer–detector system. Controller units (LDC01, MEOS, GmbH, Germany) are used to control the temperature and current settings of the laser source, hence controlling its power.

#### Optical parameter determination

Based on the experimental configuration illustrated in Fig. 2, the diffuse reflected and transmitted light from the tissue samples were measured. The tissue diffuse light was recorded using data-collecting optical fiber that is connected to a compact spectrometer (STDFSM, Touptek Photonics Co. Ltd., Zhejiang, China). The recorded measurements were then analyzed using the spectrometer’s software (ToupSpm). The optical parameters (absorption coefficients (*μ*_*a*_), reduced scattering coefficients (*μ*_*s*_*’*)) of Caco2 cells (colon carcinoma), and WI38 cells (healthy cells) were measured at 665 nm and 808 nm incident laser irradiation using Kubelka–Munk (KM) calculations. KM model assumes two coefficients (*a* and *b*) that can be related to the tissue diffuse reflected and transmitted light as follows [52]:

1 $$\mathrm a=\frac{1+\mathrm R_{\mathrm d}^2-\mathrm T_{\mathrm d}^2}{2{\mathrm R}_{\mathrm d}}$$

2 $$\mathrm b=\sqrt{\mathrm a^2-1}$$

where *R*_*d*_ and *T*_*d*_ are tissue reflectance and transmittance, respectively. Additionally, the losses in the flux due to the absorption (*A*) and the scattering (*S*) are related to the two KM coefficients (*a* and *b*) by the following formulas:

3 $$\mathrm S=\frac1{\mathrm{bd}}\ln\left[\frac{1-{\mathrm R}_{\mathrm d}(\mathrm a-\mathrm b)}{{\mathrm T}_{\mathrm d}}\right]$$

4 $$\mathrm A=\left(\mathrm a-1\right)\mathrm S$$

Furthermore, ${\mu }_{a}$ and ${\mu }_{s}$ can be calculated by:

5 $$\mathrm A=2\mu_{\mathrm a}$$

6 $$\mathrm S=\frac34\mu_{\mathrm s}\left(1-\mathrm g\right)-\frac14\mu_{\mathrm a}$$

where *μ*_*s*_ (1 − *g*) = μ’_s_ is named the reduced scattering coefficient. Finally, the total attenuation *(μ*_*t*_*)* of light that travels within tissues can be determined using Beer-Lambert law as follows:

7 $$\mathrm I={\mathrm I}_0\mathrm e^{-\mu_{\mathrm t}\mathrm d}$$

where ${I}_{0}$ is the intensity of the incident laser, $I$ is the intensity of light transmitted through the sample, $d$ is the optical path length, and the extinction coefficient ${\mu }_{t}={\mu }_{a}+{\mu }_{s}$ is also referred to as the total attenuation coefficient. Given ${\mu }_{a}$ and *μ `*_*s*_ from Kubelka–Munk calculations, and ${\mu }_{t}$ from (7), the anisotropy factor *g* can be determined.

#### Modeling the optical fluence distribution

The diffusion equation approximates the radiative transport equation (RTE) for a relatively simple description of the behavior of light transport through biological tissues [51]. The finite element method can be used to determine the fluence rate distribution at the cell line surface via solving the following diffusion equation [48, 53]:

8 $$\frac{\partial\Phi\left(\overset\rightharpoonup{\mathrm r},\mathrm t\right)}{\mathrm c\partial\mathrm t}+\mu_{\mathrm a}\Phi\left(\overset\rightharpoonup{\mathrm r},\mathrm t\right)-\nabla\left[\mathrm D\nabla\Phi\left(\overset\rightharpoonup{\mathrm r},\mathrm t\right)\right]=\mathrm S\left(\overset\rightharpoonup{\mathrm r},\mathrm t\right)$$

*D* is the diffusion coefficient which is defined as D=1/(3(µ_a_+µ'_s_)) , $\Phi\left(\overset\rightharpoonup r,t\right)$ is the fluence rate (in W/cm^2^) and $S\left(\overset\rightharpoonup r,t\right)$ is the source term (in W/cm^3^ sr).

#### Observing the fluorescent effect of the Ashwagandha chitosan NPs during cell imaging

The inverted microscope (Zeiss Axio Vert.A1; Zeiss, Gottingen, Germany) observed the *fluorescent light of* the *Ash C NPs* for the cells that were irradiated with NIR laser (665 nm) during the cell imaging.

### Therapeutic assay

**Fig. 3:**
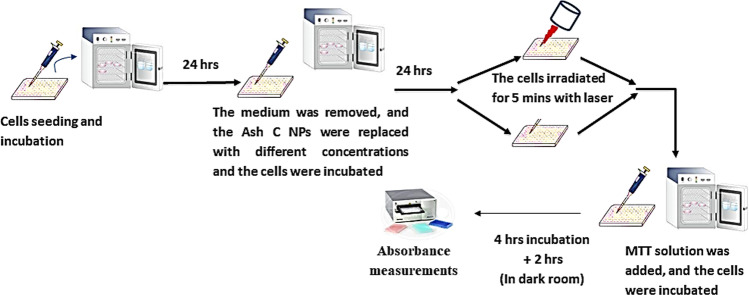
Schematic of the therapeutic assay process

The therapeutic assay evaluated the therapeutic effect of the synthesized *Ash C NPs* in two groups of treatment (absence of the NIR laser irradiation and presence of NIR at 665 nm, for 5 min in continuous mode) against colon carcinoma in vitro (Caco-2 cell line). A 3-(4,5-dimethylthiazol-2-yl)-2,5-diphenyltetrazolium bromide (MTT) assay estimated the cell viability. In brief, after the cells were maintained for 24 h in RPMI 1640 medium, the medium was detached. At that moment, the cells were washed two times at 25 °C by 1.0 M phosphate-buffered saline (PBS; pH 7.4). Then, *Ash C NPs* replaced the medium; in a range of concentrations from 6.25 to 100 μg/mL. CO_2_ incubator (5%) incubated the treated cells for 24 h at 37 °C. After adding MTT solution, the cells were re-incubated for 4 h in the CO_2_ incubator (37 °C). The medium was removed for adding 100 μL of dimethyl sulphoxide to each well. At that time, a dark room incubated the cells for 2 h. In the end, the wells’ absorbance was read with a plate reader at 570 nm (SunriseTM; Tecan Group, Männedorf, Switzerland). Schematic of the therapeutic assay process is presented in Fig. 3.

### The statistical analysis

The data were described statistically in terms of mean ± standard deviation (± SD) and standard error mean (SEM). One-way variance (ANOVA) analysis with Bonferroni post hoc multiple two-group comparisons finished the comparison mission between the two treatment groups. The ANOVA test was used because it is a technique that quantifies the predictive value of predictor in the model that uses the comparing groups of data, and the post hoc is considered a pooled variance that estimates all the used data for the particular test. All statistical analyses were performed using the computer program IBM SPSS (Statistical Package for the Social Science; IBM Corp, Armonk, NY, USA) and release 22 for Microsoft Windows. *P* values less than 0.05 were considered statistically significant.

## Results

### Nanoparticle characterization

**Fig. 4:**
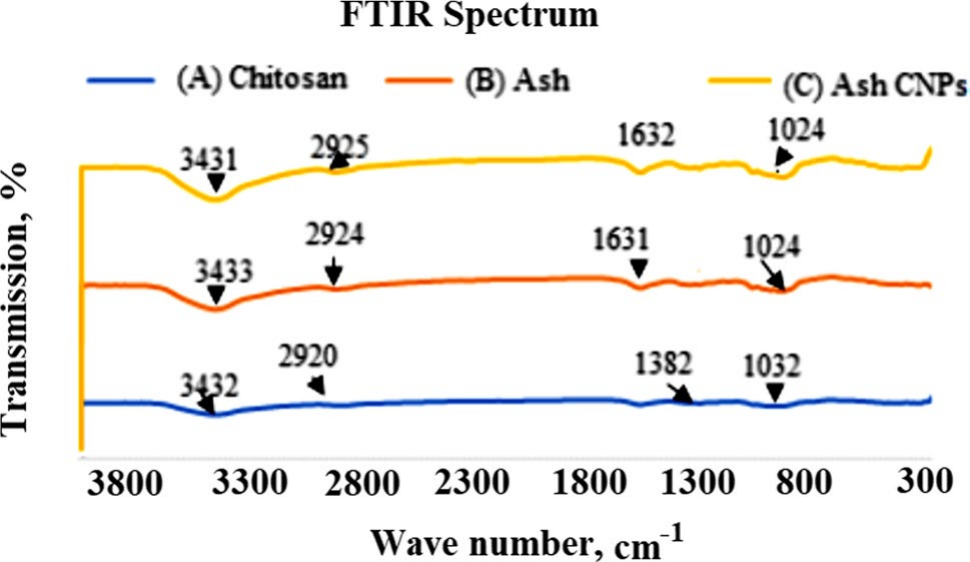
FTIR analysis of (**A**) chitosan extract, (**B**) water root extract of Ashwagandha (**C**) Ash CNPs; the scanning transmission was attuned at scanning speed 2 mm, a spectral range 4000–500 cm.^−1^

The FTIR analysis of *Ash C NPs*, Egyptian Ashwagandha root water extract (*Ash*), and chitosan analyzed the nature of chemical functional groups. Then, studying the capping ligands that control the biosynthesized polymeric nanoparticles’ stability. FTIR spectra are shown in Fig. 4. The chitosan spectrum appeared in a band in the region of 3432 to 524 cm^−1^. The Ash spectrum showed peaks at 1024, 1158, 1422, 1631, 2924, and 3433 cm^−1^. The FTIR spectrum of *Ash C NPs* presented peaks at 3431, 2925, 1632, and 1382 cm^−1^.

**Fig. 5:**
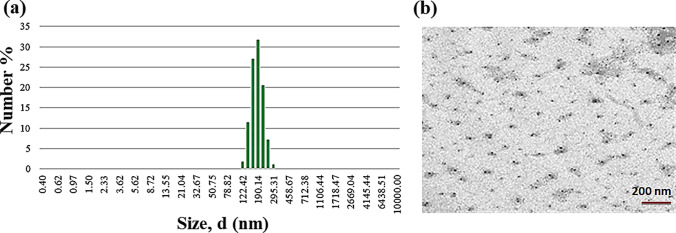
**a** DLS distributions of effective diameters for *Ash C NPs*. **b** TEM measurements for *Ash C NPs*, scale bar = 200 nm

DLS results are presented in Fig. 5a. The *Ash C NP* spectrum presented a diameter-sized distribution board ranging from ~ 100 to ~ 300 nm. The peak diameter of *Ash C NPs* is 188.3 nm. For the zeta potential (ZP) measurements, the mean surface charge, in hydro solution, of the *Ash C NPs* was 51.2 mv. Transmission electron microscope (TEM) imaging (Fig. 5b) shows stable aqueous colloids of non-aggregated spherical particles.

### Cancerous cell discrimination via Ash C NPs and the NIR laser light propagatio*n*

#### Reflectance and transmittance light measurement

**Fig. 6:**
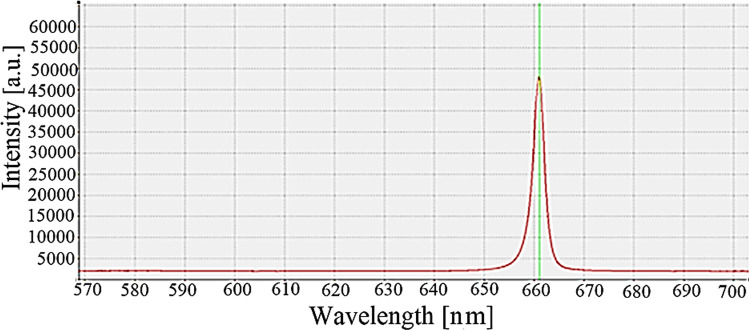
Example of the signal detected using the spectrometer at 665 nm; the signal was carried out using the A STDFSM digital fiber spectrometer

A STDFSM digital fiber spectrometer obtains the reflectance and transmittance measurements; it is linked with the computer for processing and storage. Figure 6 shows an example of the detected signal of diffuse reflectance using the spectroscopy of cells irradiated at 665 nm. The experimental study has been running with a 20° spatial step and continues to scan up to 20°. The experimental measurements for *R*_*d*_ and *T*_*d*_ were obtained five times, and the mean values with their standard deviation are presented in Table 1.

**Table 1:** The measurements of the transmittance and reflectance at 808 nm and 665 nm

Cell culture	Nanoparticles	*R*_*d*_		*T*_*d*_		*T*_*c*_	
		808 nm	665 nm	808 nm	665 nm	808 nm	665 nm
Caco2	*Ash C NPs*	0.0366 ± 0.0018	0.2526 ± 0.036	0.8606 ± 0.0046	0.6262 ± 0.075	0.3398 ± 0.0045	0.4088 ± 0.022
	Without NP injection	0.1063 ± 0.0029	0.2245 ± 0.020	0.3194 ± 0.0078	0.6935 ± 0.017	0.1515 ± 0.0017	0.3726 ± 0.019
WI 38	*Ash C NPs*	0.0111 ± 0.0028	0.3577 ± 0.018	0.3441 ± 0.0094	0.4118 ± 0.017	0.1814 ± 0.0019	0.3321 ± 0.020
	Without NP injection	0.1128 ± 0.0017	0.1250 ± 0.030	0.5977 ± 0.0585	0.6024 ± 0.025	0.2515 ± 0.0035	0.2115 ± 0.019

#### Estimation of the optical parameters

Kubelka–Munk mathematical model calculated the optical parameters (reduced scattering coefficient (*μ’*_*s*_) and absorption coefficient (*μ*_*a*_)) from the estimated values of the diffuse transmittance and reflectance. Table 2 shows the obtained results. The results show the optical parameters (*μ*_*a*_, *μ*_*s*_, *g*) of the Caco-2 and WI-38 cells. These results were estimated using the Kubelka–Munk model and Bouguer–Beer–Lambert law.

**Table 2:** Optical parameters at 665 nm and 808 nm

Wavelength (nm)	Sample	Nanoparticle	*μ*_*a*_ ${(\mathrm{cm}}^{-1})$	*μ*_*a*_ ${(\mathrm{cm}}^{-1})$	$g$
665	WI 38 cells	*Ash C NPs*	5.201	11.870	0.8238
		Without NP injection	1.727	12.07	0.755
	CaCo 2 cells	*Ash C NPs*	0.543	10.251	0.9272
		Without NP injection	4.447	14.425	0.6464
808	WI 38 cells	*Ash C NPs*	0.652	8.294	0.3381
		Without NP injection	1.603	13.927	0.7703
	CaCo 2 cells	*Ash C NPs*	0.652	8.294	0.3381
		Without NP injection	1.603	13.927	0.7703

#### Optical fluence rate distribution

**Fig. 7:**
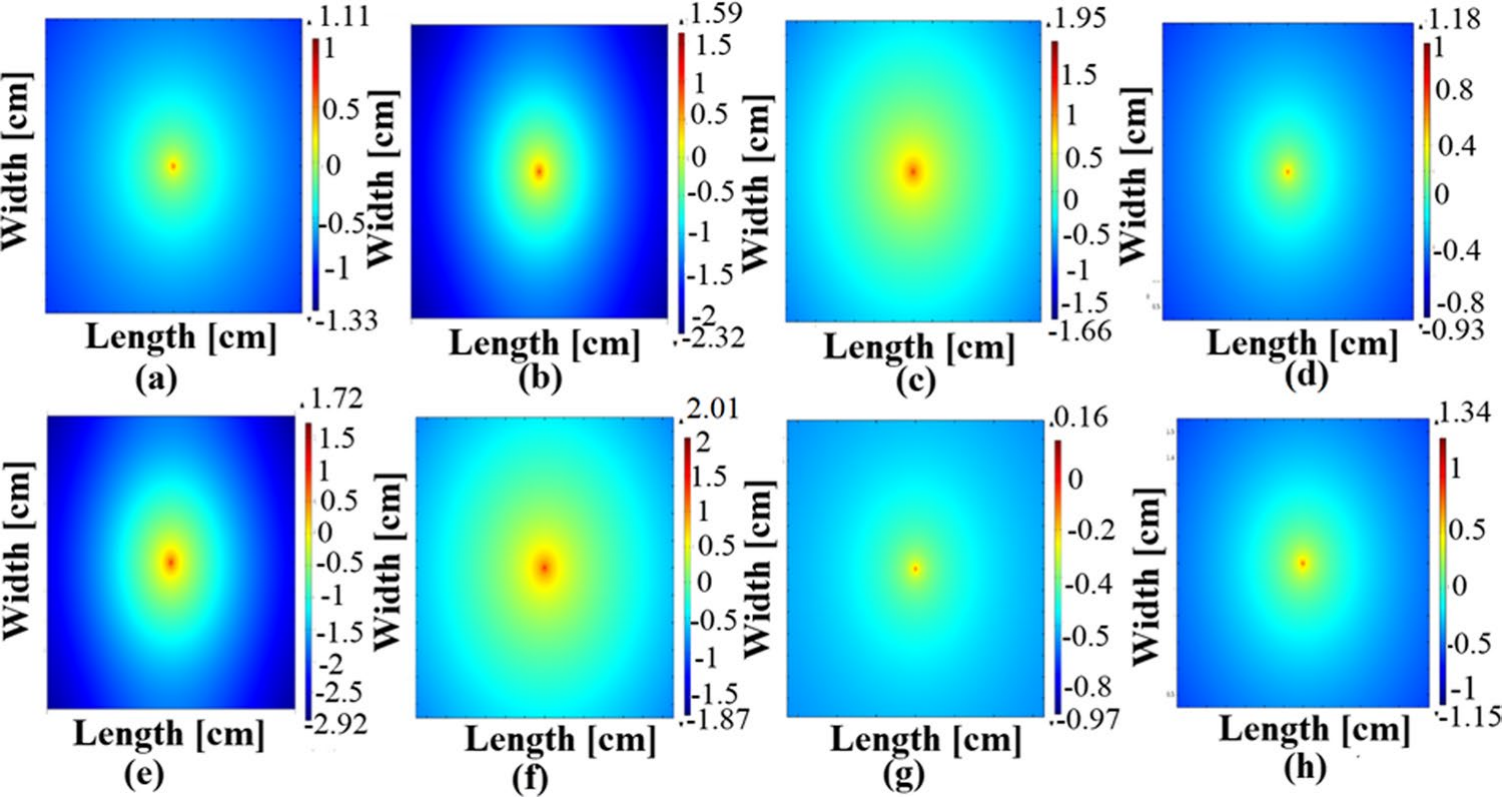
Optical fluence rate distribution. **a** WI38 cells, **b** Caco2 cells, at 665 nm, **c** WI38 cells, **d** Caco2 cells, at 808 nm, **e** Ash C NP WI38 cells, **f** Ash C NP Caco2 cells, at 665 nm, **g** Ash C NP WI38 cells, **h** Ash C NP Caco2 cells at 808 nm

The images were reconstructed to WI-38 and Caco-2 cells in two cases (the injection of NPs and non-injection of nanoparticles). The logarithmic fluence (log (Φ)) distribution images are presented in Fig. 7.

As shown in the figure, the spatial distribution of the fluence (log (Φ)) varies at each utilized wavelength and after the NP addition as well. The maximum fluence value in WI38 cells without NPs was 1.11 and 1.95 at 665 nm and 808 nm, respectively, while these values changed to 1.72 and 0.16 after adding the Ash C NPs. On the other hand, maximum fluence in Caco2 cells at 665 nm and 808 nm was 1.59, 1.18, 2.01, and 1.34 before and after adding the NPs, respectively. Moreover, the minimum fluence values have also varied before and after adding the Ash C NPs. Table 3 summarizes the obtained values in all cases.

**Table 3:** Summary minimum and maximum values of the optical fluence for each sample at the two utilized wavelengths

Wavelength (nm)	Sample	Nanoparticle	Max. log (Φ)	Min. log (Φ)
665	WI 38 cells	*Without NP injection*	1.1	− 1.33
		*Ash C NPs*	1.72	− 2.92
	CaCo 2 cells	*Without NP injection*	1.59	− 2.32
		*Ash C NPs*	2.01	− 1.87
808	WI 38 cells	*Without NP injection*	1.95	− 1.66
		*Ash C NPs*	0.16	− 0.97
	CaCo 2 cells	*Without NP injection*	1.18	− 0.93
		*Ash C NPs*	1.34	− 1.15

#### Observing the fluorescent light of Ash C NPs under the inverted microscope

**Fig. 8:**
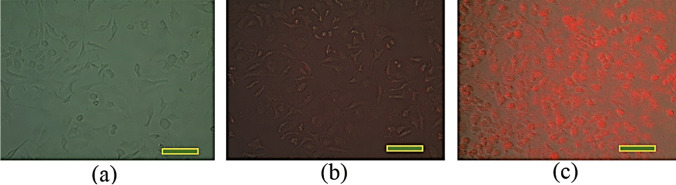
**a** Control untreated cells; **b** cells irradiated with laser and did not injected by *Ash C NPs*; **c** cells irradiated with laser and injected by *Ash C NPs* (scale bar = 20 μm)

The photomicrographs cells under the inverted microscope appeared the effect of the NIR irradiation (665 nm laser) as fluorescent light on the cells’ surface in the case of *Ash C NP* injection, as revealed in Fig. 8c.

### Therapeutic assay

**Fig. 9:**
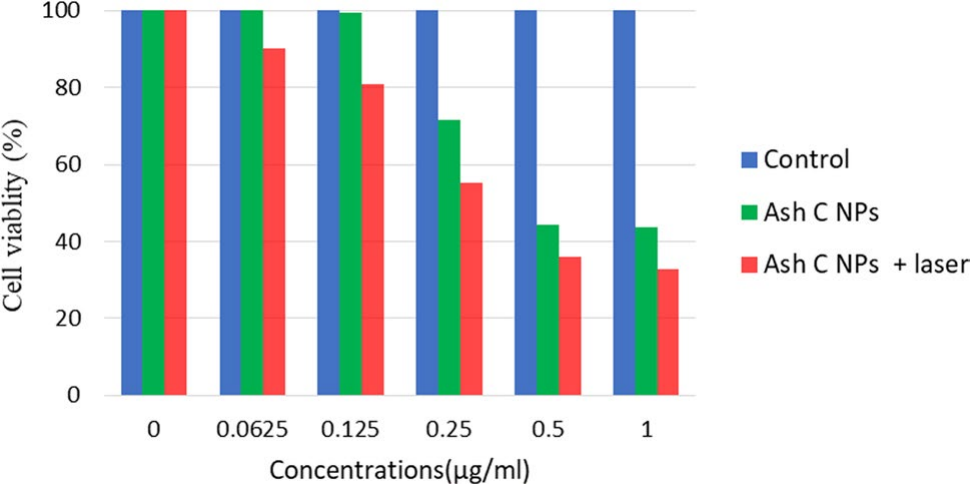
Caco2 cell viability assay versus the different concentrations of the Ash C NPs, in the presence and the absence of laser irradiation, data presented as mean ± SD. *P < 0.05 compared between the two groups

For the biomedical application, nanotechnology and NIR light are significant in cancer treatment and molecular tissue imaging because nanotechnology shows the ability of NIR in early cancer detection, which provides a guide for cancer treatment. Figure 9 shows the cell viability for the *Ash C NPs* in the absence and the presence of the laser irradiation (665 nm, 5 min) compared to control. The measured *P* value was less than 0.05.

## Discussion

The FTIR analysis of Ash C NPs, chitosan, and Egyptian Ashwagandha root water extract (*Ash*) was carried out to study the chemical functional groups and evaluate the capping ligand environment responsible for nanoparticle stability. Table 4 shows the analysis of the chitosan, *Ash*, and *Ash C NP* spectra. A strong band 3432–524 cm^−1^ region in the chitosan spectrum indicates N–H and O–H stretching. All bands that were detected in the chitosan spectrum were conveyed by Fernandes Q M [27] and Ibitoye E B [54]. The presented peaks of the Ashwagandha root extract spectrum were reported by Trivedi team [55]. The formulation of *Ash C NPs* is based on the chemical interaction between the nitro compound group (chitosan) and the stretching vibration of OH (Ash), as described by Nagaonkar, D. [14].

**Table 4:** FTIR spectra analysis

FT-IR peak spectra (cm^−1^)			Vibrational mode
Chitosan	Ash	Ash C NPs	
1382		1382	δs (CH3) in NHCOCH3 group
1032	1024	1024	(C–O) in secondary OH group
2920	2924	2925	C-H alkanes stretching
	1158		C-O stretching of carboxylic acid
1631	1631	1632	C-O secondary amide stretch
	1422		C-H alkanes bending (δ(CH2) in CH2OH group)
3432	3433	3431	Symmetric stretching vibration of OH

The mean hydrodynamic radii of the nanomaterials were delivered by DLS, which shows a beak at the spectrum, indicating no particle aggregation. *Ash C NPs* recorded a high stability level because they show strong positive zeta potential (ZP) above 30 mV, indicating the cationic surface charge stability [56]. High values (above 30 mV) of ZPs reflect dispersion stability [24] and a low level of nanoparticle aggregation, as reported by Wang Y [17].

The TEM characterization showed regular contours of *ASh C NPs*. The results agree with the zeta potential measurements where the high stable NPs indicate regular nanoparticle contours [57]. The TEM images appear a decrease in the measured nanoparticles’ diameters compared to DLS measurements because of the shrinking dry state, as Singh R [48] reported. The DLS measurements show larger nanoparticle sizes than the TEM measurements because of the light scattering effect of the DLS instrument, which causes increasing in the metric sizes, as reported by Souza et al. [58].

Different optical parameter values of the Caco2 cells and WI38 cells injected with the Ash C NPs were obtained for the diagnostic assay. This difference results from the variation in the diffuse transmittance and reflectance of Caco 2 cells (colon carcinoma) compared to the healthy tissue (WI38 cells). Additionally, the estimated parameters using 665 nm dramatically changed in the case of NP injection compared to the cells irradiated with 808 nm. The fluence rate distribution of the healthy cells differs in the cancerous cells with and without NP injection to both wavelengths (808 nm and 665 nm). The untreated control cells and the non-injected cells with NPs were irradiated with laser, and they did not appear as shining light on the cells’ surface, while the injected cells with *Ash C NPs* appeared as a shining light on the cells’ surface during exposure to NIR irradiation.

For the therapeutic assay, for 24 h, the Caco 2 cells were treated using *Ash C NPs* in different concentrations. Figure 9 revealed the concentration versus the cell viability of the synthesized *Ash C NPs* in the presence and absence of NIR. *Ash C NP* concentration is inversely proportional to the cell viability. The survival in the case of laser irradiation was decreased compared to non-laser irradiation. The *P* values were less than 0.05, and it is considered statistically significant when the two treatment groups were compared. Such observation agrees with Yi XM [59], who evaluates NIR irradiation consequences in cancer management.

This finding agrees with Asrar et al. [33] who proved the photothermal therapy’s effectiveness by using nanotubes (CNTs) and titanium dioxide (TiO_2_) nanoparticles against the melanoma tumor. Behnam et al. [34] studied the PTT effect of the silver nanoparticles combined with polyethylene glycol and the oxide carbon nanotubes under the effect of near-infrared irradiation (808 nm), and he proved that those NPs destroyed the malignant melanoma tumors through the PTT technique. Marpu, S.B [15] and Abuelmakarem [60] reported the effectiveness of employing chitosan in forming contrast agents for clinical imaging and photonic application. This represented research approves the results of Agrawal, P. [18], who employed the chitosan NPs in molecular imaging due to their ability to emit and absorb the NIR laser, which penetrates several centimeters of the tissue.

Additionally, the recorded difference in the transmittance and the reflectance measurements, the optical parameters, and the fluence distribution between the different studied samples is considered an approach for cancer diagnosis. Therefore, the development of optical biomedical imaging techniques is greatly interested in tissue probing to precisely predict photon propagation trajectories and the fluence rate distribution within irradiated tissues. The presented study avoided using all harmful materials encapsulated with nanoparticles for therapeutic and imaging purposes. Consequently, our natural composite is a suitable candidate for in vivo application in preoperative localization of cancer as well as during intra-operative management, where the chitosan NPs are accumulated in the cancer tissue more than they do in the normal tissue due to the enhanced permeability and retention (EPR) effect.

## Conclusion

The present study evaluated the *Ash C NPs* in cancer therapy and diagnosis. A stable *Ash C NPs* were synthesized successfully in a nanoform using ionic gelation methods and were characterized using the TEM, DLS, and FTIR. The FTIR analysis showed that the interaction between the *Ash C NPs* is based on the chemical interaction between the nitro compound group (chitosan) and the OH (Ash) stretching vibration. Strong positive zeta potential (ZP) above 30 mV indicates cationic surface charge stability. A digital fiber spectrometer attained the transmittance and reflectance measurements of the cells exposed to 808 nm and 665 nm. The diffusion equation has measured the light propagation on healthy and cancerous cell lines. The results presented a difference in the transmittance and reflectance measurements between the normal and the cancer cells. Photomicrograph images representing optical fluence distribution at the cells’ surface provided differentiation between cancer and normal cells. The synthesized NPs shined a light on the photographed cells during laser exposure. Moreover, the survival rate was decreased in the presence of the laser radiation when MTT carried it out. This research article represents a simple technique for cancer theranostic based on the optical tissue properties and the fluorescent *Ash C NPs*.
